# Human Macrophages and Dendritic Cells Can Equally Present MART-1 Antigen to CD8^+^ T Cells after Phagocytosis of Gamma-Irradiated Melanoma Cells

**DOI:** 10.1371/journal.pone.0040311

**Published:** 2012-07-02

**Authors:** María Marcela Barrio, Riad Abes, Marina Colombo, Gabriela Pizzurro, Charlotte Boix, María Paula Roberti, Emmanuelle Gélizé, Mariana Rodriguez-Zubieta, José Mordoh, Jean-Luc Teillaud

**Affiliations:** 1 Centro de Investigaciones Oncológicas, Fundación Cáncer FUCA, Buenos Aires, Argentina; 2 INSERM UMR S 872, Paris, France; 3 Centre de Recherche des Cordeliers, Université Pierre et Marie Curie – Paris6, UMR S 872, Paris, France; 4 Université Paris Descartes, UMR S 872, Paris, France; 5 Instituto de Investigaciones Bioquímicas de Buenos Aires, CONICET, Fundación Instituto Leloir, Buenos Aires, Argentina; Université Paris Descartes, France

## Abstract

Dendritic cells (DC) can achieve cross-presentation of naturally-occurring tumor-associated antigens after phagocytosis and processing of dying tumor cells. They have been used in different clinical settings to vaccinate cancer patients. We have previously used gamma-irradiated MART-1 expressing melanoma cells as a source of antigens to vaccinate melanoma patients by injecting irradiated cells with BCG and GM-CSF or to load immature DC and use them as a vaccine. Other clinical trials have used IFN-gamma activated macrophage killer cells (MAK) to treat cancer patients. However, the clinical use of MAK has been based on their direct tumoricidal activity rather than on their ability to act as antigen-presenting cells to stimulate an adaptive antitumor response. Thus, in the present work, we compared the fate of MART-1 after phagocytosis of gamma-irradiated cells by clinical grade DC or MAK as well as the ability of these cells to cross present MART-1 to CD8^+^ T cells. Using a high affinity antibody against MART-1, 2A9, which specifically stains melanoma tumors, melanoma cell lines and normal melanocytes, the expression level of MART-1 in melanoma cell lines could be related to their ability to stimulate IFN-gamma production by a MART-1 specific HLA-A*0201-restricted CD8^+^ T cell clone. Confocal microscopy with Alexa Fluor®^647^-labelled 2A9 also showed that MART-1 could be detected in tumor cells attached and/or fused to phagocytes and even inside these cells as early as 1 h and up to 24 h or 48 h after initiation of co-cultures between gamma-irradiated melanoma cells and MAK or DC, respectively. Interestingly, MART-1 was cross-presented to MART-1 specific T cells by both MAK and DC co-cultured with melanoma gamma-irradiated cells for different time-points. Thus, naturally occurring MART-1 melanoma antigen can be taken-up from dying melanoma cells into DC or MAK and both cell types can induce specific CD8^+^ T cell cross-presentation thereafter.

## Introduction

Cutaneous melanoma (CM) accounts for 4% of all neoplasia and it is the tumor with the fastest growing incidence worldwide [1]. Melanoma tumors are highly resistant to chemotherapy, but more responsive to immunological treatments. A large variety of antigens have been associated to CM, such as Melan A/MART-1 [2], [3], gp100 [4], Tyrosinase [5], TRP-2 [6], and NY-ESO-1 [7]. MART-1 is a hydrophobic transmembrane protein without glycosylation sites highly enriched in early melanosomes. MART-1 is necessary for gp100 function, another antigen associated to CM, involved in the regulation of melanosome formation [8]. MART-1 is expressed in skin and retinae melanocytes and in the majority of melanoma tumors, but it is absent from other tissues and tumors. It was isolated thanks to the specific recognition by T lymphocytes of MART-1 derived peptides, specially in the context of the HLA-A0201 haplotype, present in tumor infiltrates from melanoma patients [2], [3]. Thus, MART-1 is immunogenic in humans and has been widely exploited to induce anti-melanoma immunity in patients by means of several vaccination strategies. Among them, the use of MART-1 peptides either injected with adjuvants and/or pulsed on DC has been tested in clinical settings, although with very modest outcomes so far [9]–[11]. Also, MART-1 specific immune responses are frequently assessed to monitor the ability of melanoma vaccines to induce immunity in treated patients [12].

Using whole irradiated tumor cells to load DC could be preferable to develop DC-based vaccines since melanoma cells could contribute with known antigens such as MART-1 and probably unknown antigens. We have used this strategy to vaccinate melanoma patients with a mixture of gamma-irradiated melanoma cell lines and BCG, a potent inflammatory adjuvant [13], and plus GM-CSF to further attract DC to the vaccination site [14]. We and others have demonstrated in murine models [15], and in humans [16], [17] that when DC engulf gamma-irradiated melanoma cells, antigens can be cross-presented for the generation of HLA class I/peptide-complexes, allowing the induction of specific CTLs. However, in the human, the fate and immunogenic potential of DC that have phagocytosed dying tumor cells or their debris remains an open issue.

The use of irradiated allogeneic tumor cells is based on the paradigm that tumor cells would only trigger MHC-restricted tumor-specific immunity after being phagocytosed by DC, the main initiators of immune response able to activate naïve CD8^+^ T cells [18]. After phagocytosis, DC evolve to a mature phenotype, diminish their phagocytic ability, express HLA class II and co-stimulatory molecules on their surface, and acquire the capacity to present antigens in the appropriate self-HLA context. In parallel, and through the expression of adequate chemokine receptors, such as CCR7, DC are able to migrate to the draining lymph nodes to prime naive lymphocytes and trigger cellular and/or humoral immunity [19]. Both CD4^+^ and CD8^+^ naïve cells can be primed by DC [18]. CD8^+^ T cells can be stimulated after phagocytosis by a process called cross-presentation that allows exogenous peptides to be presented in the context of HLA Class I molecules [20]. Some other studies have used non-naturally occurring tumor-associated antigens such as tumor cells virally transduced with antigens or apoptotic tumor cells infected with recombinant viruses encoding melanoma associated antigens [21]–[24]. However, few of these studies have evaluated the cross-presentation of naturally occurring melanoma antigens taken up from apoptotic/necrotic tumor cells.

Other phagocytes like macrophages could also be possible mediators in the induction of antitumor immunity, although their clinical use has focused only on their tumoricidal activity. Notably, the ability of human macrophages prepared for clinical use to cross-present naturally-occurring tumor-associated antigens has not been investigated, although it has been shown that thioglycolate-elicited peritoneal or bone-marrow derived mouse macrophages can present efficiently an exogenous antigen (OVA-beads or OVA-derived peptide) and stimulate naive CD8^+^ T cells to differentiate into OVA-specific memory cells [25], [26] Brayer et al also showed that thioglycolate-elicited peritoneal exudate mouse macrophages exhibit an enhanced cross-presentation of influenza hemaglutinin (HA) and OVA-derived peptides with targeted STAT3 disruption [27]. It has also been shown that thioglycolate-elicited peritoneal mouse macrophages are more efficient in inducing cross-presentation following phagocytosis than when loaded with OVA-derived peptide [28]. More recently, it has been shown that lymph node resident CD169^+^ mouse macrophages are responsible for early activation of OVA-antigen specific CD8^+^ T cells [29]. Thus, there is no demonstration that clinical grade human macrophages can efficiently cross-present naturally-occurring tumor antigens and, if so, how it compares with the well-documented cross-presentation achieved by human DC prepared for therapeutic use. Of note, when mice were vaccinated with DC loaded with apoptotic/necrotic B16 cells (DC-Apo/Nec), a tertiary lymphoid structure was generated at the vaccination site that contained a wide variety of cell populations, including macrophages, polymorphonuclear cells, as well as CD4^+^ and CD8^+^ T lymphocytes found together with DC [30]. This suggests that macrophages could also contribute to the antitumor response against gamma-irradiated tumor cells locally, or after migration to the lymph nodes.

Thus, we tested the cross-presentation of human clinical grade DC and macrophages cultured with gamma-irradiated melanoma cells as a source of MART-1 antigen to a specific CD8^+^ T cell clone. Using a new mouse high-affinity monoclonal antibody (mAb) against MART-1, 2A9, we analyzed the presence of MART-1 within two different human antigen-presenting cells, DC and IFN-gamma activated macrophages (MAK), after phagocytosis of MART-1 expressing gamma-irradiated melanoma cells at different time points. We also related this antigen capture to MART-1 cross-presentation to specific CD8^+^ T lymphocytes.

## Materials and Methods

### Tumor cells

Six melanoma cell lines (MEL-XY1, MEL-XY3, MEL-XX4 [17], MEL-XX5, MEL-XY6, and MEL-XY10) were established from tumor biopsies from metastatic melanoma patients at the Instituto Alexander Fleming (Buenos Aires, Argentina). Jau cells are melanoma cells derived from a metastatic tumor biopsy that can be cultured for several days *in vitro* (primary culture). All cell lines and Jau cells, except MEL-XX4 and MEL-XY10, are HLA*0201 positive as determined by FACS analysis with a phycoerythrine (PE)-labeled anti-HLA-A2 monoclonal antibody (mAb) (clone BB7.2, BD Biosciences, San José, CA, USA). All tumor biopsy samples were obtained from melanoma patients who were enrolled in several clinical trials authorized by the Comité de Docencia e Investigación of Instituto Alexander Fleming (Institutional Review Board) and who gave written informed consent. Cells were cultured as previously described [17].

MART-1 non-expressing breast carcinoma MCF-7 cells [31] [obtained from the American Tissue Culture Collection (ATCC, Manassas, USA)] were cultured in DMEM/F12, supplemented with 100I.U./mL penicillin, 100 µg/mL streptomycin, 2 mM glutamine, 10 µg/mL insulin, 10% FBS. HLA-A*0201 positive MART-1 non-expressing breast carcinoma IIB-BR-G cells were cultured as previously described [32]. MART-1 non-expressing HT-29 colon carcinoma cells were obtained from the ATCC and cultured in DMEM supplemented with 2 mM L-glutamine, 3.5 mg/mL sodium carbonate, 4.5 g/L glucose and 10% FBS.

### Macrophages and dendritic cells

Human IFN-gamma activated macrophages (termed Macrophage Activated Killer, MAK) were a kind gift of Dr J. Bartholeyns (Immuno-Designed Molecules S.A., Paris, France). They were obtained as previously described [33].

Human DC were derived from PBMC of buffy-coats obtained from healthy donors at the Hemotherapy Department of the Instituto Alexander Fleming as previously described [17]. This procedure received approval from the Comité de Docencia e Investigación of the Instituto Alexander Fleming and healthy donors gave written informed consent. To induce DC maturation, 2 µg/mL LPS (Lipopolysaccharide from *E. coli* J5, Sigma, St. Louis, MO, USA) were added and the cells were further cultured for 48 hours. DC maturation was assessed by CD80, CD83 and CD86 labelling.

MAK and DC phenotypes have been extensively characterized elsewhere [17], [33]. They were both obtained under GMP conditions identical to those used for clinical trials [34], [35].

### Anti-MART-1 monoclonal antibodies

MAbs directed against MART-1 were obtained by immunizing BALB/c ByJ mice (Charles River Laboratories, L'abresle, France) with purified glutathione-S-transferase (GST)-MART-1 fusion obtained by cloning MART-1 cDNA isolated from the IIB-MEL-J melanoma cell line [36] into the pGEX-2T vector (GE Healthcare, Saclay, France). After ELISA screening for anti-MART-1 IgG mAbs, two hybridomas, 1F9 and 2A9, were selected and cloned as previously described [37]. 1F9 and 2A9 mAbs are IgG1, κ as determined by isotype-specific ELISA. Purification of these antibodies was performed by Protein-A affinity chromatography (rProtein A Sepharose™ fast flow) (GE Healthcare). For confocal microscopy experiments, the purified 2A9 mAb was coupled to Alexa Fluor ^647^ fluorochrome using the Alexa Fluor ^647^ Protein Labeling Kit (Molecular Probes, Eugene, OR, USA). Animal used for hybridoma production were handled in compliance with Institutional guidelines and experiments were approved by the Ethics Committee in Animal Experiment Charles Darwin, Paris, France.

The affinity constants (K_aff_) of 1F9 and 2A9 mAbs for GST-MART-1 coated onto ELISA plates were determined using the protocol described by Beatty *et al.* [38]. Briefly, 96-well ELISA plates (Nunc, Roskilde, Denmark) were coated overnight at 4°C with four concentrations (4, 2, 1 and 0.5 µg/mL) of GST-MART-1 in PBS. Serial three-fold dilutions (from 1.33×10^−7^ down to 2.26×10^−12^ M) of purified 1F9, 2A9 mAbs or of the anti-MART-1 A103 mAb (Calbiochem-Merck Chemicals, Beeston, United Kingdom) [39] were then performed in duplicate for each concentration of GST-MART-1. After incubation for 2 hours at room temperature, binding was revealed using Alkaline Phosphatase (AP)-conjugated goat anti-mouse IgG (Fc specific) antibodies (1∶1,000) (Southern Biotechnologies, Birmingham, AL, USA) for 2 hours at room temperature. O.D._405 nm_ was measured after 30 minutes incubation at room temperature.

### Competition binding assay

A competition assay was performed by ELISA using biotinylated 2A9 mAb and unlabelled 1F9, 2A9 or A103 anti-MART-1 mAbs as competitors. 96-well ELISA plates (Nunc) were coated with 5 µg/mL purified GST-MART-1 in PBS. Three-fold dilutions of purified 1F9, 2A9 or A103 mAbs in PBS-Tween 0.05% (starting at 20 µg/mL) were then incubated for 2 hours at room temperature. After washing, biotinylated 2A9 mAb was added (700 ng/mL in PBS-Tween 0.05%) and incubated for 2 hours at room temperature. After washing, microplates were incubated with a streptavidin-horse radish peroxidase (HRP) solution (Dako, Glostrup, Denmark) for 20 minutes at room temperature. O.D._450 nm_ was measured after 30 minutes incubation at room temperature.

### Immunohistochemistry

Melanoma, colon and breast carcinoma biopsies were obtained from the Instituto Alexander Fleming and from the Pathology Department, Hospital Interzonal General de Agudos (HIGA-Eva Perón), Buenos-Aires, Argentina. Immunohistochemistry (IHC) anonymised sections from melanoma biopsies were also kindly provided by Pr. Vacher-Lavenu (Department of Pathology of the Tarnier Unit of Dermatopathology, Cochin Hospital, Paris, France). Obtention of tumor biopsy samples was authorized by the Comité de Docencia e Investigación of Instituto Médico Especializado Alexander Fleming, in which case patients gave written informed consent. In case of biopsies from HIGA-Eva Perón and from Cochin Hospital, since these samples belong to the Hospital archives and are anonymous, patient's consent was not requested by the Bioethics Committee of the HIGA-Eva Perón and the Ethics Committee from Cochin Hospital. Biopsy specimens were fixed in 10% neutral buffered formalin, embedded in paraffin and 4-µm tissue sections were cut and processed for IHC. Epitope retrieval was performed by incubation in a pressure cooker or in a water bath with citrate buffer, pH 6.0, for 1 minute after reaching boiling temperature or at 95°C for 40 minutes, respectively. After inactivation of endogenous peroxidase, tissue sections were blocked with normal horse serum (Vectastain Elite ABC Kit; Vector Laboratories, Burlingame, CA, USA) or with 5% human AB serum (Biowest, Nuaillé, France) for 30 min, and incubated overnight at 4°C with 0.5 µg/mL 2A9 mAb. After washing, slides were incubated with biotinylated universal secondary antibodies (Vectastain Elite ABC Kit) or with biotinylated sheep IgG anti-mouse IgG1 antibodies (The Binding Site, Birmingham, United Kingdom), followed either by avidin-biotin complex reagent and peroxidase substrate (VECTOR NovaRed Substrate Kit; Vector Laboratories) or by streptavidin-HRP (Dako) followed by AEC substrate (AEC Substrate kit for Peroxidase, Vector Laboratories). Counterstaining was performed with 10% hematoxylin. As a negative control, primary antibodies were omitted or a mouse isotype control [IgG1, κ (Southern Biotechnologies)] was used.

### Western Blot of melanoma cell extracts

Melanoma cells were lysed with 50 mM Tris-HCl (pH 7.5), 5 mM EDTA, 150 mM NaCl, 1% NP-40, 0.1% SDS, in presence of Protease Inhibitor Cocktail (Sigma) for 30 minutes on ice. Protein extracts were obtained after centrifugation at 8,000×*g* for 10 minutes at 4°C and total protein concentration was measured by the Bradford assay. Fifty micrograms protein were fractionated in 12% SDS-PAGE gels, transferred onto nitrocellulose membranes (Sigma), blocked with 3% dried skim milk in PBS and then incubated with 2A9 mAb (5 µg/mL) and an anti-β-actin mAb (clone AC-15) (Sigma) overnight at 4°C. Binding of the mAbs was revealed using Alkaline Phosphatase (AP)-conjugated goat F(ab)′_2_ anti-mouse IgG(H+L) (Jackson Immunoresearch, West Grove, PA, USA). Color development substrate was 5-bromo-4-chloro-3-indolyl-phosphate/nitro blue tetrazolium (NBT/BCIP) (Promega).

### MART-1 detection by FACS and confocal microscopy

MART-1 was detected by staining melanoma cells with 2A9 mAb, after fixation with 3% paraformaldehyde (PFA) (Fluka, Buchs, Switzerland) and permeabilized with 0.05% saponin (PBS-Sap). 2A9 mAb or an isotype-matched control antibody (Sigma) (5 µg/mL) were incubated with cells for 1 hour at 4°C in PBS-Sap. Cells were washed and further incubated with 1/50 secondary R-PE-conjugated F(ab′)_2_ goat-anti-mouse Ig(H+L) antibodies (Dako) for 1 hour at 4°C. They were analyzed by flow cytometry with a FACSCalibur (BD Biosciences) with the CellQuestPro software (BD Biosciences). Results are indicated as percentage of positive cells and Mean Fluorescent Intensity (MFI).

For confocal microscopy, cells were grown on 0.1 mg/mL poly-L-lysine (Sigma) treated coverslips (Knittel Gläser KN0011879, Braunschweig, Germany) for 2–3 days at 37°C in a 5% CO_2_ incubator. After washing, cells were fixed, permeabilized as above and incubated with Alexafluor^647^ coupled 2A9 mAb (2 µg/mL in PBS-Sap) for 1 hour on ice. After washing, coverslips were mounted onto glass slides with Mowiol solution (Calbiochem-Merck Chemicals) or with Prolong Gold with Hoechst (Invitrogen Life Technologies). As controls for the phagocytosis assay, co-cultures were incubated at 4°C instead of 37°C. Slides were analyzed with a confocal microscope LSM 510 (Carl Zeiss, Jena, Germany). Pictures were processed with the AIM or the Zen 2008 Light Edition software (Carl Zeiss).

### Induction of IFN-gamma production by co-culture of melanoma cells and M27 T cell clone

3×10^5^ MEL-XY3 melanoma cells and M27 cells were co-cultured (1∶1) in 600 µL of AIM-V medium (Invitrogen), in 24-well plates, overnight at 37°C. The M27 HLA-A*0201-restricted anti-MART-1 (AAGIGILTV) CTL clone was kindly provided by Dr. C. Yee (Fred Hutchinson Cancer Research Centre, Seattle, WA, USA). M27 cells were cultured and expanded as previously described [40].

Cells were then harvested, centrifuged, and supernatants collected. Culture supernatants were tested for IFN-gamma content with BD OptEIA human IFN-gamma ELISA set (BD Biosciences), following the manufacturer's recommendations. A calibration curve was performed for each experiment and the sample concentration was calculated by log-log regression analysis using Cembal 2.2 software [17].

### Phagocytosis assay

Gamma-irradiated melanoma MEL-XY3 cells (gamma-MEL-XY3) were generated by 70 Gy gamma irradiation (Siemens Accelerator, Instituto Alexander Fleming) and frozen under liquid N_2_ until use. Apoptosis was assessed by Annexin-V/PI staining and FACS analysis (BD Biosciences). For MAK phagocytosis experiments, 50 µL of gamma-MEL-XY3 cells (2×10^6^/mL) were deposited onto poly-L-lysine-treated lamellae into a well of a 24 well-tissue culture plate. The same volume of MAK cells [6×10^6^/mL] was immediately added and mixed with gamma-MEL-XY3 cells (MAK:gamma-MEL-XY3 cell ratio: 3∶1). After 20 minutes incubation at room temperature, 150 µL of 5% FBS-Iscove's Modified Dulbecco's Medium (IMDM Medium, Invitrogen Life Technologies)] were added and cells were incubated at 37°C for different periods of time (1, 3, 6, 24, and 48 hours). Either MAK cells or MEL-XY3 cells were also deposited on coverslips to be used as controls in labeling experiments.

Immature DC (iDC) from 5 day-cultures or LPS-maturated DC (mDC) were harvested and placed [3×10^5^/mL] into a 24-well tissue culture plate with AIM-V medium. Gamma-MEL-XY3 cells were added at a DC/MEL-XY3 ratio of 3∶1 for different times (1, 3, 6, 24, and 48 hours). In each case, after incubation, cells were washed with cold PBS, blocked with PBS-0.1% BSA and stained either with the anti-CD86-Fluorescein Isothiocyanate (FITC) (clone B70/B7-2, BD Biosciences) (DC) or with a mixture of anti-CD14-FITC (clone MΦP9)/anti-CD32-FITC (clone FLI8.26) (BD Biosciences) (MAK).

Cells were then washed, fixed and permeabilized before staining with Alexafluor^647^-2A9 mAb as described above and analyzed by confocal microscopy. Controls included DC and MAK incubated with Alexafluor^647^-2A9 mAb, or gamma-MEL-XY3 cells incubated with anti-CD86-FITC or a mixture of anti-CD14/anti-CD32-FITC mAbs.

### Cross-presentation assay

Cross-presentation of MART-1 after DC/MAK phagocytosis was tested using the M27 HLA- anti-MART-1 T cells. DC were obtained from highly purified (>98%) CD14^+^ monocytes derived from a HLA A*0201 donor using anti-CD14 microbeads (Miltenyi Biotec, Germany). Monocytes were differentiated to iDC by 5 days culture as previously described [17]. HLA A*0201 DC or MAK (3×10^5^) were incubated with 10^5^ gamma-MEL-XY3, or gamma-MEL-XY10, or gamma-IIB-BR-G as a negative control, for 3, 6, 24 and 48 hours. Co-cultures were then incubated overnight with M27 T cell clone (10^5^ cells) in AIM-V medium. IFN-gamma amounts in co-culture supernatants were determined in triplicate as described above. Positive controls included LPS-treated mDC loaded for 3 hours at 37°C with 20 µg/mL MART-1 or gp100 peptides plus 3 µg/mL β2-microglobulin or MEL-XY3 live cells. Other controls included M27 cells incubated alone or with gamma-MEL-XY3 or gamma-IIB-BR-G at 3, 6, 24 and 48 hours post-irradiation, and with DC or MAK.

### Statistics

Comparisons between IFN-gamma basal levels (M27 clone+gamma-irradiated tumor cells) and co-culture-stimulated M27 cells (either with DC:gamma-MEL-XY3 or MAK:gamma-MEL-XY3) were analyzed using Student's t-test to determine the p-values. p<0.05 was considered significant.

## Results

### Characterization of anti-MART-1 2A9 mAb

**Figure 1:**
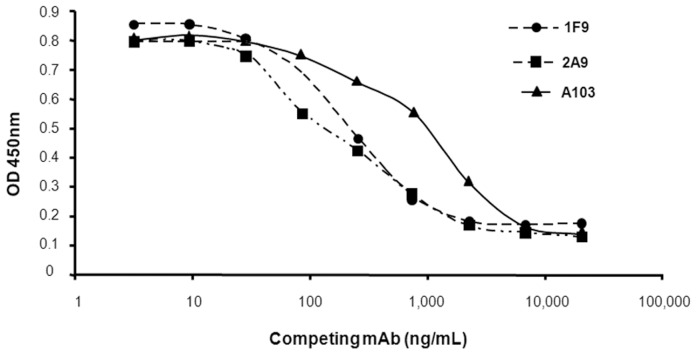
Competition ELISA between anti-MART-1 1F9, 2A9 and A103 mAbs and biotinylated-2A9 mAb. 96-well ELISA plates were coated with purified GST-MART-1 and incubated with purified 1F9, 2A9 or A103 mAbs (three-fold dilutions in duplicate). Biotinylated-2A9 mAb was then added. A streptavidin-HRP solution was used as detection reagent. Results shown correspond to one representative experiment out of three independent assays.

1F9 and 2A9 mAbs that bound to GST-MART-1 fusion protein in ELISA were selected. Competition experiments performed with GST-MART-1 coated onto ELISA plates and biotinylated 2A9 showed that both antibodies efficiently compete for MART-1 binding, as well as the A103 mAb (**Figure 1**), an antibody previously shown to be a binder of a MART-1 epitope (100–110) located within the C-terminus part of the molecule [41]. Thus, these mAbs recognize the same epitope or overlapping epitopes located in the C-terminus part of MART-1. They also exhibited higher affinity for the GST-MART-1 fusion protein than the A103 mAb, in the nanomolar range, as shown using the ELISA method developed by Beatty et al [38] (**Table 1**).

**Table 1:** Kaff determination of anti-MART-1 mAbs.

Antibody	K_aff_ * (M^−1^)
1F9	1.32±0.39×10^9^
2A9	0.26±0.16×10^9^
A103	3.2±1.7×10^7^

* Mean of 24 values (n = 4 independent experiments).

**Figure 2:**
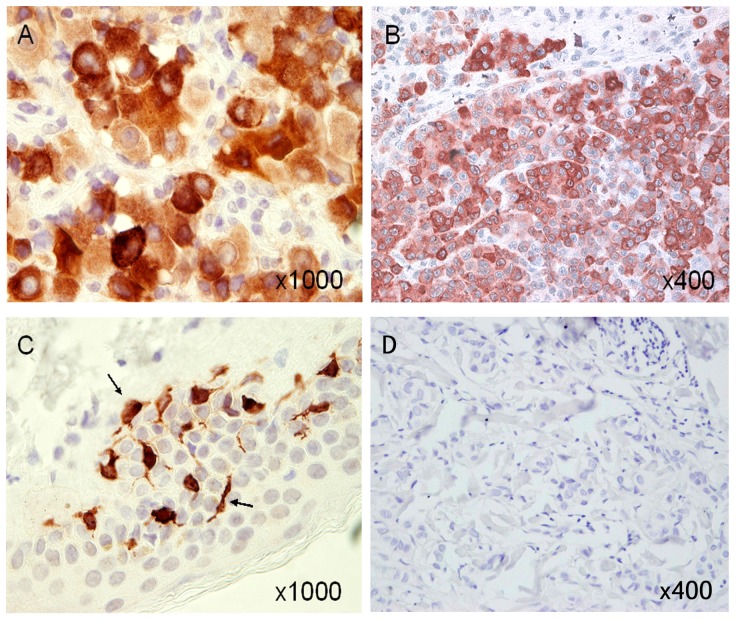
Immunohistochemical staining of melanoma tumor tissues with the 2A9 mAb. 2A9 mAb staining of a primary tumor and of one metastasis from the same melanoma patient is shown in (A) and (B), respectively; staining of melanocytes present in epidermis is shown in (C), while no staining of keratinocytes is observed. Black arrows indicate melanocytes. No staining is observed with a breast carcinoma tumor biopsy (D). Original magnification: A and C = 1000×; B and D = 400×.

Since the 2A9 mAb exhibited the highest affinity and could be biotinylated without losing its ability to bind MART-1, as opposed to 1F9 mAb, all the other experiments were performed with this antibody. The 2A9 mAb stained MART-1^+^ melanoma cells using indirect IHC. A strong cytoplasmic staining of melanoma cells could be observed, with discrete punctuate structures in the cytosol being detected. It stained tumor cells both from primary tumor (**Figure 2A**) and metastases (**Figure 2B**), as well as melanocytes present in epidermis (**Figure 2C**). No staining of keratinocytes (**Figure 2C**) and breast cancer cells (**Figure 2D**) was detected. Similarly, the 2A9 mAb did not stain colon carcinoma cells (not shown).

### Melanoma cells with different MART-1 expression elicit different IFN-gamma production by MART-1 specific M27 T cells

**Figure 3:**
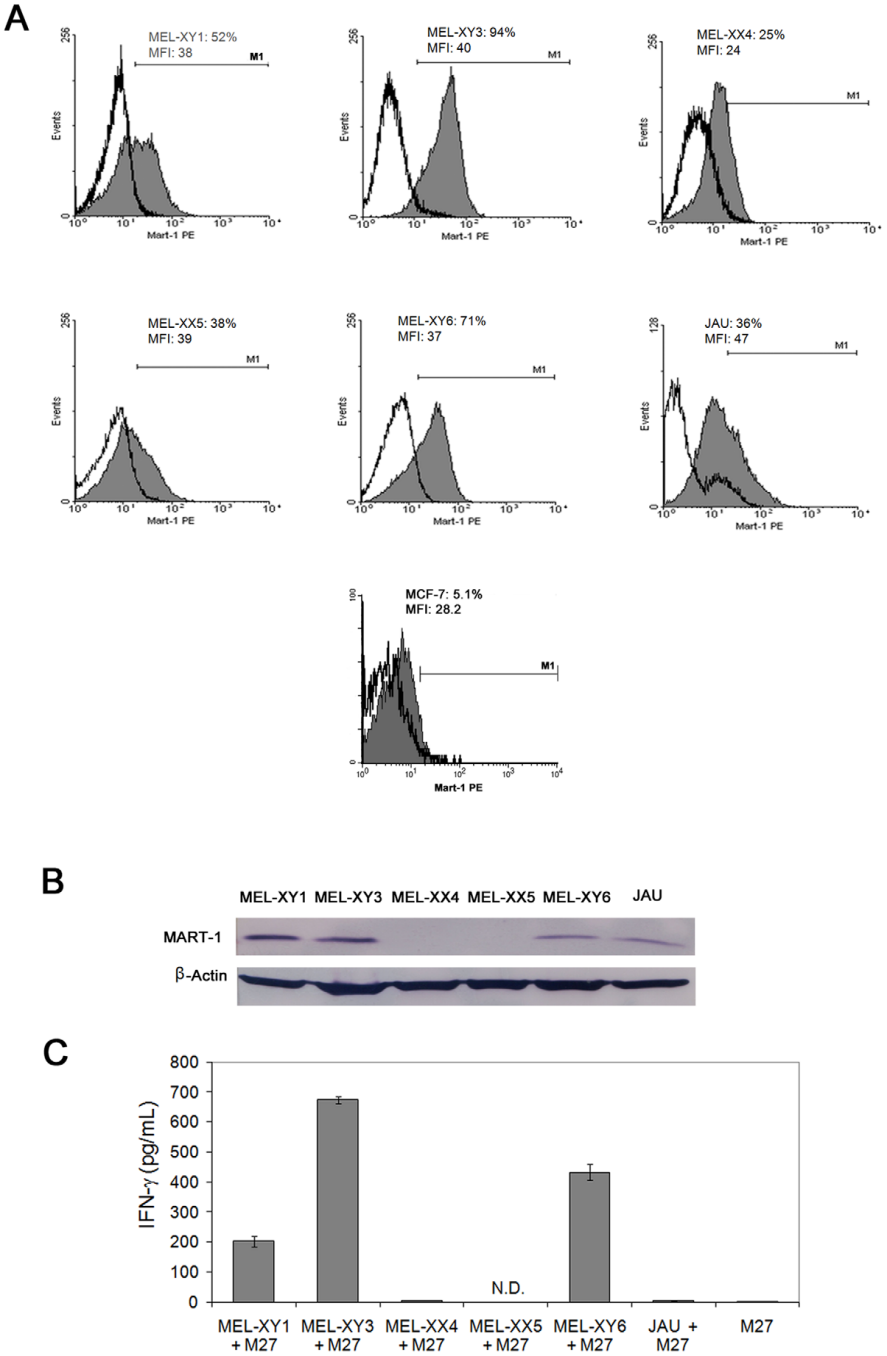
Differential MART-1 expression in melanoma cells and production of IFN-gamma by M27 CD8^+^ T cells. (A) Intracellular immunofluorescence was performed with cells from five melanoma cell lines, in melanoma cells from a primary culture (JAU), and MCF7 breast carcinoma cells. Empty histograms show isotype controls and filled histograms staining with the 2A9 mAb. Percentage of positive cells and MFI are indicated. (B) The expression of MART-1 in cells from different melanoma cell lines was tested by Western blotting with the 2A9 mAb, using an anti-β-actin mAb as a loading control. (C) IFN-gamma production by MART-1-specific M27 T cell clone. Melanoma cells and M27 cells were co-cultured overnight at a 1∶1 ratio. Culture supernatants were then tested by ELISA for IFN-gamma content in triplicates. The mean IFN-gamma values (pg/mL ±S.D.) are indicated. Results are representative of three independent experiments.

FACS analysis after intracellular immunofluorescence staining by the 2A9 mAb showed that cells from five different melanoma cell lines and cells from a primary culture of melanoma (JAU) differently express MART-1 (**Figure 3A**). MEL-XY3 cells strongly express MART-1. MEL-XY6 and MEL-XY1 cells also markedly express MART-1, but are heterogeneous as the percentage of stained cells is lower than that of MEL-XY3. Of note, only 35–40% JAU and MEL-XX5 cells were stained. Finally, MEL-XX4 cells showed the lowest level of MART-1 expression, both in terms of percentage of labeled cells and MFI. The breast carcinoma MCF-7 cells did not exhibit any staining (**Figure 3A**). As shown in **Figure 3B**, MART-1 could also be detected by Western blotting, showing that the 2A9 mAb binds a linear epitope. MART-1 was detected in MEL-XY1, MEL-XY3, MEL-XY6 and Jau melanoma cell extracts but not in MEL-XX4 and MEL-XX5 cell extracts. These latter cells exhibited the lowest percentage of labeled cells in the immunofluorescence assay (**Figure 3A**). Finally, MEL-XY1 and MEL-XY3 cells showed the highest level of MART-1 expression. MART-1 expression in MEL-XY6 was lower, although these cells were readily stained (**Figure 3A**).

The melanoma cell lines were then tested for their ability to induce IFN-gamma production by the MART-1 specific HLA-A*0201-restricted M27 T cells. As shown in **Figure 3C**, cells that express MART-1 (MEL-XY1, MEL-XY3, and MEL-XY6) elicited a HLA-A*0201-restricted IFN-gamma production. Of note, all these cells express HLA-A*0201 to a similar level (data not shown). Interestingly, cells that showed the highest percentage of labeled cells in the 2A9 intracellular immunofluorescence assay (MEL-XY3 and MEL-XY6) elicited the highest IFN-gamma production. As expected, MEL-XX4 cells did not induce any IFN-gamma production since these cells are negative for HLA-A*0201 expression (not shown).

No M27 IFN-gamma secretion was observed with the JAU primary culture cell. This could be due to the fact that only 36% of these cells express MART-1 and that, in addition, only a low HLA-A*0201 expression is observed in no more than 60% of the cells (not shown).

### MART-1 can be detected in human dendritic cells and macrophages after phagocytosis of gamma-irradiated melanoma cells

**Figure 4:**
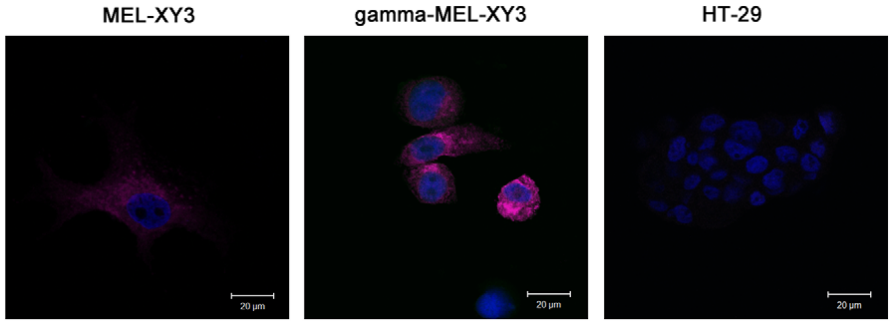
Intracellular MART-1 detection with 2A9-Alexafluor^647^ mAb by confocal microscopy. (A) MEL-XY3 viable cells and (B) gamma-irradiated MEL-XY3 cells (gamma-MEL-XY3) showed a punctuate pattern of MART-1 expression, presumably staining melanosomes, while HT-29 colon carcinoma (C) showed no staining. For better visualization, nuclei were stained with Hoechst.

We selected MEL-XY3 cells for further experiments since they exhibited higher MART-1 expression almost uniformly (>90%). First, permeabilized live and gamma-MEL-XY3 were labeled with the 2A9-Alexafluor^647^ mAb. A characteristic punctuate intracellular pattern of MART-1 expression was detected with the antibody in both live and gamma-MEL-XY3 (**Figure 4** **, left and middle panels**). HT-29 colon carcinoma cells were negative (**Figure 4, right panel**).

**Figure 5:**
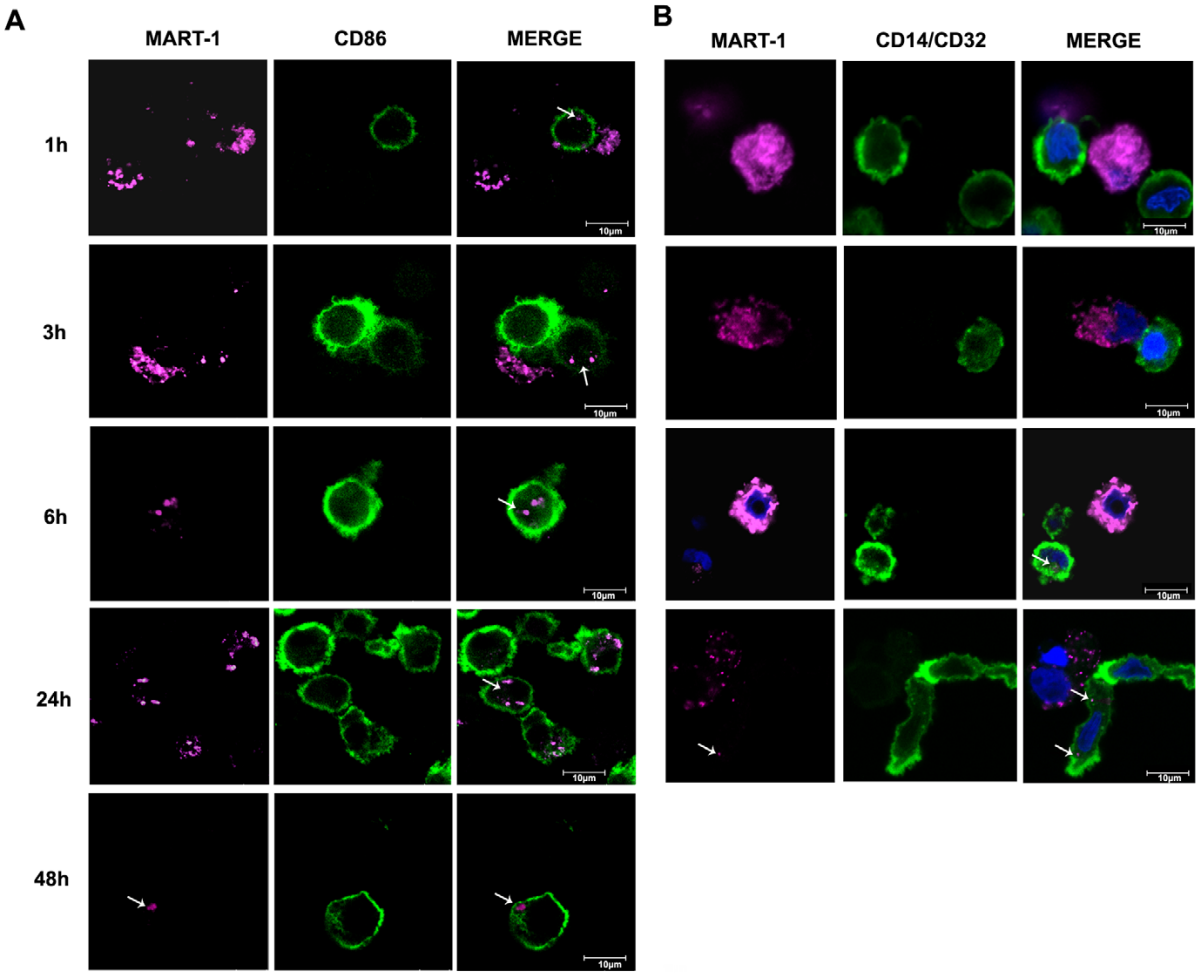
Detection of MART-1 in DC and MAK after phagocytosis of gamma-irradiated MEL-XY3 cells. Co-cultures between gamma-MEL-XY3 cells and DC (A) or MAK (B) and cell labeling and confocal microscopy were performed as described under Materials and Methods. White arrows show MART-1 labeled material within DC and MAK.

We then examined whether MART-1 could be detected into phagocytes after phagocytosis of gamma-MEL-XY3 cells by MAK or iDC. Gamma-MEL-XY3 cells are a mixture of apoptotic and necrotic cells as determined by Annexin V and propidium iodide staining (**Figure S1**). As shown in **Figure 5A**, the 2A9-Alexafluor^647^-labeled cell fragments from gamma-irradiated MEL-XY3 cells could be detected attached to the cell surface and inside iDC from 3 hours up to at least 48 hours after initiation of co-cultures. We estimated the proportion of DC that have captured some MART-1 labeled material from total DC (% phagocytosis) to be 10–20% of cells by counting several pictures (at least 150 cells/time point) (not shown). Lack of phagocytosis was observed when LPS-mDC were tested in the phagocytosis assay (<3% phagocytosis) (not shown). When MAK were tested in the phagocytosis assay (**Figure 5B**), labeled gamma-irradiated MEL-XY3 cells were seen in close attachment to the MAK surface at earlier time points (1–3 hours) and 2A9-Alexafluor^647^-labeled material could be observed inside MAK cells from 3 hours and up to 24 hours after the initiation of co-cultures. Around 10–15% of MAK exhibited intracellular MART-1 pink labeling after a 6 hours period of co-culture. This percentage tends to decrease thereafter, down to around 5% at 24 hours. MAK co-cultures were analyzed for only 24 hours since after that time they started dying (not shown). No phagocytosis was observed when DC or MAK co-cultures were incubated at 4°C (not shown).

### DC and MAK are able to cross-present to specific CD8^+^ T cells MART-1 captured from gamma-irradiated melanoma cells

Since MART-1-labeled material could be detected inside phagocytes from 3 hours up to 48 hours after phagocytosis of gamma-MEL-XY3 cells, we then investigated whether MART-1 could be cross-presented to specific CD8**^+^** T cells at different times after phagocytosis.

**Figure 6:**
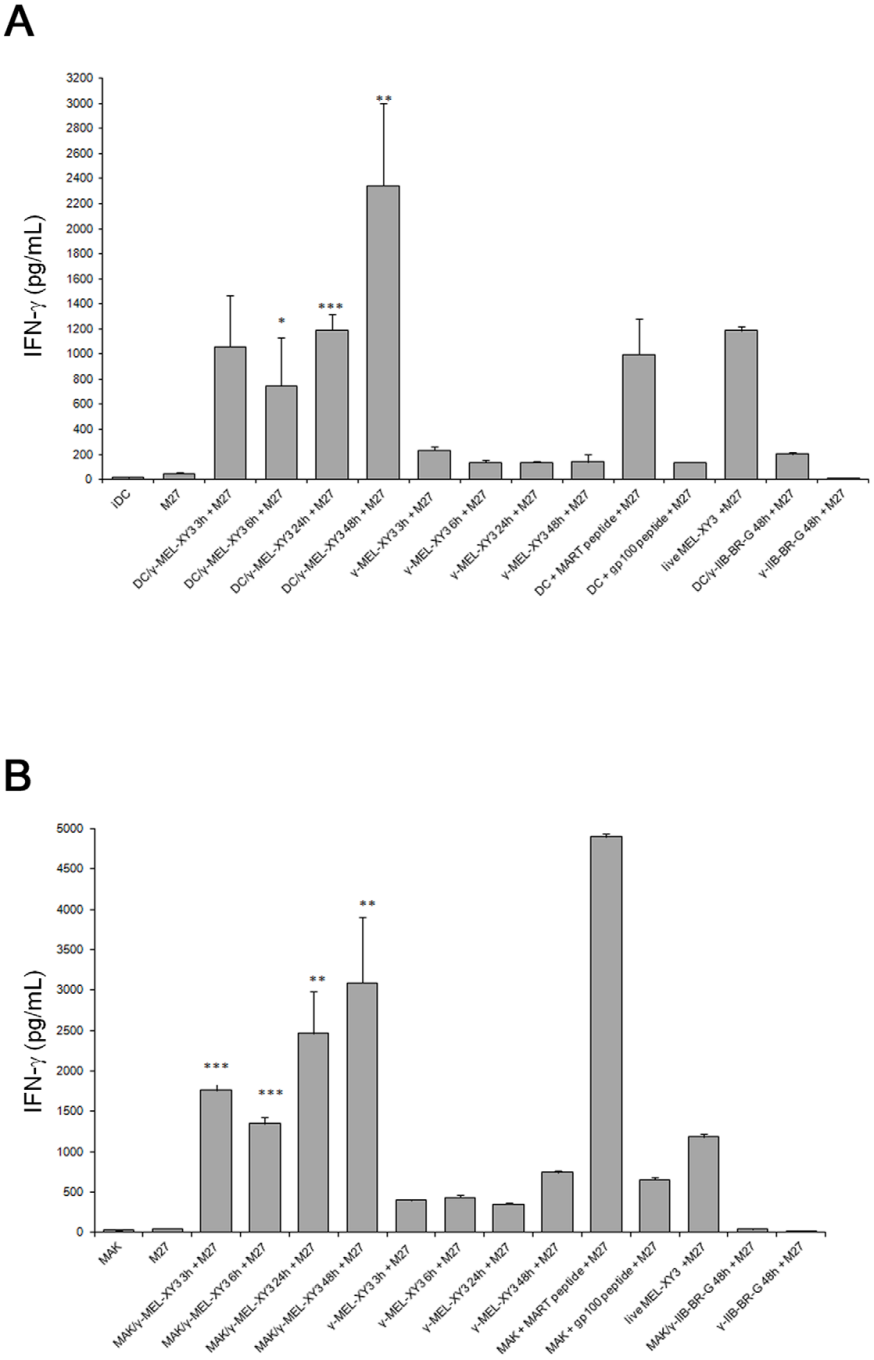
MART-1 cross-presentation to M27 CD8^+^ T cell clone. Cross-presentation assay was performed as described under Materials and Methods. For co-cultures, a 3∶1 ratio iDC (panel A) or MAK (panel B) to gamma-MEL-XY3 target cells was used and incubation was done for the indicated times. After co-culture, M27 CD8^+^ T cells were added and cells were further incubated overnight. Positive controls were either live MEL-XY3 cells or MART-1 peptide loaded mDC or MAK, incubated with M27 cells overnight. As negative controls, gp100 peptide or IIB-BR-G cells were used. MAK, DC and gamma-MEL-XY3 were also tested alone and with M27 cells to measure basal IFN-gamma levels. Results are expressed as mean ±SD pg/mL IFN-gamma released into the supernatant. One representative experiment performed in triplicate is shown. Differences between basal levels (M27 clone+gamma-irradiated tumor cells) and co-culture-stimulated M27 cells (either with DC:gamma-MEL-XY3 or MAK:gamma-MEL-XY3) were statistically significant from 3 to 48 hours (* p<0.05; ** p<0.005; *** p<0.0005).

As observed in **Figure 6**, both DC and MAK were able to induce IFN-gamma production from M27 cells as early as 3 hours and up to 48 hours after initiation of co-cultures with gamma-MEL-XY3 cells. In both cases, the amount of IFN-gamma increased along the co-culture time, suggesting that M27 T cells are being stimulated more efficiently after longer periods of antigen processing. However, depending on the MAK co-culture experiments (n = 8), the production of IFN-gamma by MAK peaked at 24 hours and decreased at 48 hours in two experiments, likely due to a parallel decrease in MAK viability at that time. Of note, when the MART-1 expressing HLA-A*0201 negative MEL-XY10 cells were tested, a strong production of IFN-gamma by the M27 T cell clone was observed after 48 hours of co-culture with DC or MAK (data not shown), indicating that the cross-presentation observed with both DC and MAK cells is not due to a transfer of peptide loaded onto HLA-A*0201 from melanoma cells to APC. Gamma-MEL-XY3 cells induced only low IFN-gamma secretion from M27 cells between 3–48 hours after irradiation when directly incubated with these latter cells. This was expected as we previously showed that HLA-A*0201 molecules are progressively lost from the cell surface after irradiation, impairing gamma-MEL-XY3 ability to directly stimulate phagocytes with time [17]. Specific HLA-A*0201-restricted responses were obtained using either LPS–matured DC loaded with the corresponding peptide or M27 stimulation with live MEL-XY3 cells. As expected, a lack of response was observed when an unrelated gp100 peptide was used. Also, only basal level of IFN-gamma was detected when MART-1 non-expressing HLA-A*0201 positive IIB-BR-G breast carcinoma cells were used (**Figure 6A and 6B**).

## Discussion

The generation of a CD8^+^ T cell antitumor response requires the presentation of antigenic peptides on antigen-presenting cells after capture of antigens at the periphery and migration to the draining lymph nodes [18]. It needs both naïve CD4^+^ and CD8^+^ T cells to be recruited and stimulated by antigen-presenting cells. This is achieved by DC that have the extraordinary ability to stimulate naïve CD8^+^ T cells through a process called cross-presentation [20] in addition to recruit naïve CD4^+^ T cells. Thus, immunotherapeutic trials have focused on the manipulation of DC to trigger an antitumor immune response [42]. Different vaccination strategies, ranging from *ex vivo* loading of peptides onto DC followed by reinfusion [43], [44] to *in vivo* targeting of DC with antibodies [45]–[47] or subunits of toxins [48] have been developed to achieve specific immunity in preclinical models and clinical studies [11], [15], [17]. Among such strategies one consists in the ex-vivo loading onto autologous DC of doxorubicin-treated [16] or gamma-irradiated melanoma cells as a source of multiple antigens [15], [17], [33]. We have previously shown that human iDC loaded with a mixture of apoptotic/necrotic gamma-irradiated melanoma cells for 48 hours are able to cross-present MART-1 and gp100 antigens and achieved DC maturation comparable to LPS stimulation [17]. Several reports have suggested that apoptotic cells may be critical in processing antigens for cross-presentation, probably by pre-selection of immunologically important antigenic determinants [49], [50]. Besides, necrotic death is accompanied with the release of cell fragments and of a variety of molecules that could trigger an inflammatory microenvironment and give maturation stimuli to DC [51].

Other immunotherapeutic trials have been based on the use of macrophages. Notably, MAK, i.e., IFN-gamma activated macrophages derived from purified peripheral monocytes [35] have been tested in various clinical settings [52], [53]. Although macrophages are also potent antigen-presenting cells, their clinical use has focused on their short-term tumoricidal activity. In particular, the ability of human macrophages prepared for clinical use to cross-present naturally occurring tumor-associated antigens has not been investigated. Thus, in the present work, we analyzed whether phenotypically well-defined [32] clinical grade human macrophages prepared ex vivo can cross-present naturally occurring tumor-associated antigen (MART-1) after phagocytosis of apoptotic/necrotic melanoma cells and how they compare with clinical grade DC [33]. Melanoma cell death through gamma-irradiation occurs by induction of apoptosis and secondary necrosis, since after 70 Gy irradiation dying cells consist of a mixture of A+/PI− and A+/PI+ cells, as we have shown here for gamma-MEL-XY3 cells (**Fig. S1**). Firstly, using a high affinity mAb specific for MART-1, 2A9 that detects MART-1 by IHC, immunofluorescence and Western Blot in melanoma cells, the capacity of HLA-A2^+^ tumor cells to directly stimulate MART-1 specific CD8 T cells could be related to the expression level of MART-1. Secondly, after coupling to Alexafluor^647^, the 2A9 mAb was used to follow MART-1 fate in gamma-irradiated MEL-XY3 cells after co-culture with either DC or MAK. MART-1 labeled material derived from dying cells could be detected both within DC and MAK at different times after the initiation of co-cultures. No MART-1 could be detected in co-cultures incubated at 4°C, indicating that MART-1^+^ cell fragments were captured by an active phagocytosis process (not shown). Thirdly, both DC and MAK could cross-present MART-1, inducing similar levels of IFN-gamma production by a HLA-A*0201 restricted CD8 T cell clone after different times of phagocytosis. In these assays, gamma-MEL-XY3 cells alone did not present MART-1 significantly because, as previously shown [17], HLA-A*0201 molecules are progressively lost from the tumor cell surface during the gamma irradiation-induced cell death. Overall, clinical-grade MAK could cross-present MART-1 as efficiently as DC after phagocytosis of dying melanoma cells, even after a short contact period (3 hours), based on an in vitro IFN-gamma production read-out. Whether these cells could also act as antigen cross-presenting cells in vivo upon re-infusion remains to be established, as the activation with IFN-gamma makes these cells short-lived [32] and it is unclear whether they could migrate via afferent lymphatic vessels to secondary lymphoid structures to prime naive T cells. However, we have recently found evidence that, in mice vaccinated with DC loaded with apoptotic/necrotic B16 cells that achieve 80% protection against B16 challenge, a tertiary lymphoid structure is found at the vaccination site, with the presence of CD4^+^ and CD8^+^ T lymphocytes together with DC as well as macrophages [30]. Thus, one can hypothesize that, in the human setting, a similar situation could be found. Local macrophages and DC could be able to phagocyte dying tumor cells present in the vaccines, process tumor derived antigens and cross-present them to specific CD8^+^ T cells. Both DC and macrophages could contribute to the generation of an antitumor immune response. Furthermore, when BCG and GM-CSF are used as adjuvants [14], the potent inflammatory stimulus that is induced may increase the presence of macrophages at the site of vaccination. Thus, both DC and macrophages loaded with antigens derived from dying cells could potentially contribute to the induction of tumor-specific immunity either by priming naive T cells in the draining lymph nodes and/or by priming effector/memory T cells in repeatedly vaccinated patients.

## Supporting Information

Figure S1 **Annexin V and propidium iodide staining of gamma-MEL-XY3 cells.** Live MEL-XY3 cells (A) and gamma-MEL-XY3 cells (B) at 3, 6, 24, and 48 hours after irradiation were stained with Annexin V and propidium iodide (PI) and analyzed by flow cytometry as described under Materials and Methods. Early apoptotic cells were defined as Annexin V-FITC^+^/PI^−^, while necrotic cells were double-positive. 30,000 cells were analyzed in each case and percentage of early apoptotic and necrotic cells are indicated in each quadrant. A representative experiment is shown.
(TIF)
