# Supplementary figures and images for: Human Macrophages and Dendritic Cells Can Equally Present MART-1 Antigen to CD8+ T Cells after Phagocytosis of Gamma-Irradiated Melanoma Cells
Source: PLoS One. 2012 Jul 2;7(7):e40311. doi: 10.1371/journal.pone.0040311 (PMC3388056; doi:10.1371/journal.pone.0040311)

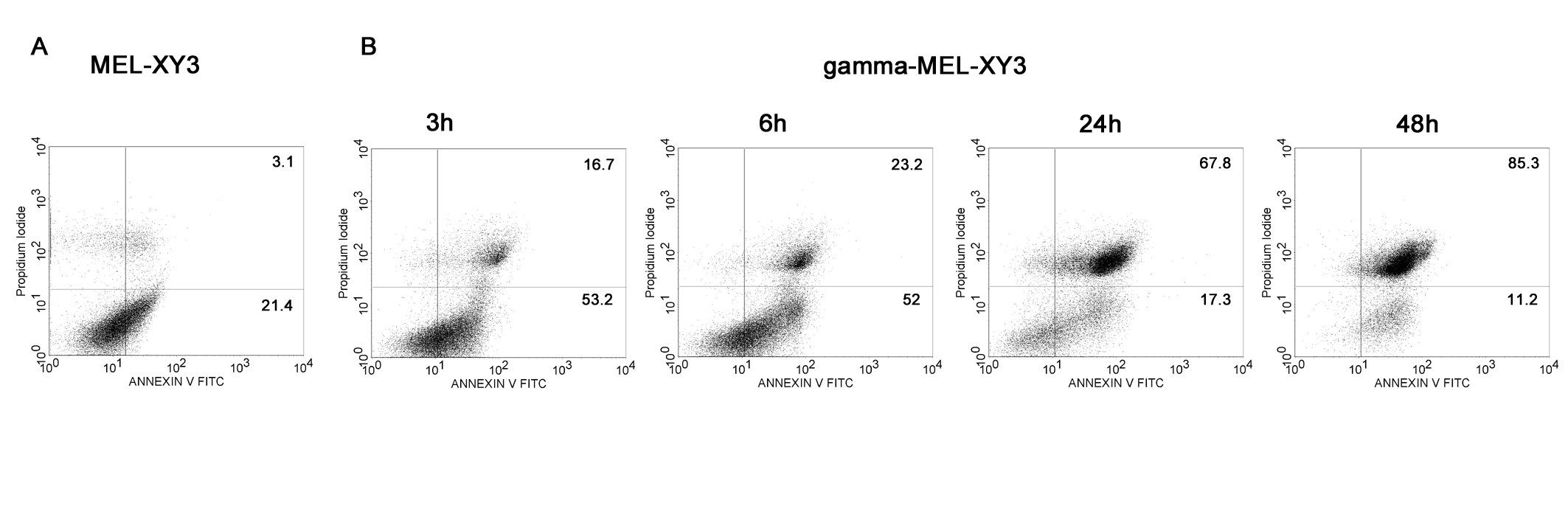

Supplement: Figure S1 — Annexin V and propidium iodide staining of gamma-MEL-XY3 cells. Live MEL-XY3 cells (A) and gamma-MEL-XY3 cells (B) at 3, 6, 24, and 48 hours after irradiation were stained with Annexin V and propidium iodide (PI) and analyzed by flow cytometry as described under Materials and Methods. Early apoptotic cells were defined as Annexin V-FITC+/PI−, while necrotic cells were double-positive. 30,000 cells were analyzed in each case and percentage of early apoptotic and necrotic cells are indicated in each quadrant. A representative experiment is shown. (TIF) [file pone.0040311.s001.tif]
